# Improvement of Quality and Disease Resistance for a Heavy-Panicle Hybrid Restorer Line, R600, in Rice (*Oryza sativa* L.) by Gene Pyramiding Breeding

**DOI:** 10.3390/cimb46100639

**Published:** 2024-09-25

**Authors:** Haipeng Wang, Gen Wang, Rui Qin, Chengqin Gong, Dan Zhou, Deke Li, Binjiu Luo, Jinghua Jin, Qiming Deng, Shiquan Wang, Jun Zhu, Ting Zou, Shuangcheng Li, Yueyang Liang, Ping Li

**Affiliations:** State Key Laboratory of Crop Gene Exploration and Utilization in Southwest China, Rice Research Institute, Sichuan Agricultural University, Chengdu 611130, Chinawanggen200109@163.com (G.W.); 15308568925@163.com (R.Q.); gong1cheng1qin@163.com (C.G.); 17378589832@163.com (D.Z.); lideke0109@163.com (D.L.); lbj415523784@163.com (B.L.); jinjinghua1984@126.com (J.J.); dengqmsc@163.com (Q.D.); sqwangscau@163.com (S.W.); zhujun987@126.com (J.Z.); zouting@sicau.edu.cn (T.Z.); lisc926105@163.com (S.L.)

**Keywords:** heavy panicle, iR600, grain quality, blast and bacterial blight resistance

## Abstract

The utilization of heavy-panicle hybrid rice exemplifies the successful integration of architectural enhancement and heterosis, which has been widely adopted in the southwest rice-producing area of China. Iterative improvement in disease resistance and grain quality of heavy-panicle hybrid rice varieties is crucial to promote their sustainable utilization. Here, we performed a molecular design breeding strategy to introgress beneficial alleles of broad-spectrum disease resistance and grain quality into a heavy-panicle hybrid backbone restorer line Shuhui 600 (R600). We successfully developed introgression lines through marker-assisted selection to pyramid major genes (*Wx^b^* + *ALK^A-GC^* + *Pigm* + *Xa23*) derived from three parents (Huanghuazhan, I135, I488), which significantly enhance grain quality and confer resistance to rice blast and bacterial blight (BB). The improved parental R600 line (iR600) exhibited superior grain quality and elevated disease resistance while maintaining the heavy-panicle architecture and high-yield capacity of R600. Moreover, the iR600 was crossed with male sterility line 608A to obtain a new heavy-panicle hybrid rice variety with excellent eating and cooking quality (ECQ) and high yield potential. This study presents an effective breeding strategy for rice breeders to expedite the improvement of grain quality and disease resistance in heavy-panicle hybrid rice.

## 1. Introduction

China is the leading producer and consumer of rice (*Oryza sativa* L.), with over 60% of the Chinese population relying on rice as staple food, while approximately 40% of the country’s total calorie intake is derived from rice [1]. The continuous advancement of rice breeding techniques over the past few decades has resulted in a sustainable enhancement in both the yield and quality of rice [2]. In the history of rice breeding, three milestone innovations in breeding methods have contributed to the improvement of rice yield: dwarf rice breeding, hybrid rice breeding, and super rice breeding [3]. However, the escalating challenges to rice production stem from the relentless population growth, diminishing availability of arable land and water resources, frequent occurrence of natural disasters, and mounting ecological pressures [4]. These challenges could be addressed through the utilization of ideotype breeding and precise molecular design, accelerating the improvement of hybrid parents by pyramiding superior alleles and cultivating new varieties of super rice with enhanced yield, quality, and resistance [5,6].

Heavy-panicle hybrid rice varieties are extensively cultivated in the southwestern region of China, making a significant contribution to ensuring food security in China. Due to the climatic characteristics of deficient solar radiation, high humidity, and small temperature difference, developing heavy-panicle hybrid rice is an effective way to increase rice yield in southwest China [7]. Heavy-panicle hybrid varieties exhibit the advantages of possessing large panicles, rapid grain filling, high seed setting rate, reduced ineffective tillering, enhanced photosynthetic efficiency, robust stalks, and increased resistance to lodging [8]. In the past few decades, a wide range of heavy-panicle hybrid rice varieties, including D-you527, Xie-you527, F-you498, Chuannong-you498, Te-you600, and Chuangu-you600 are extensively cultivated in southwest China. Despite the remarkable advancements in breeding heavy-panicle hybrid rice over the past few decades, the current varieties of this type have gradually manifested issues such as varietal homogeneity, decline in disease resistance, and poor grain quality. Therefore, it is imperative to breed heavy-panicle hybrid rice varieties with superior grain quality and enhanced disease resistance to promote their sustainable utilization.

Rice quality is usually divided into direct or indirect evaluation of processing quality, appearance quality, and ECQ, among which ECQ is the core of rice quality. The quantification of ECQ typically involves the assessment of amylose content (AC), determination of gelatinization temperature (GT), and evaluation of gel consistency (GC). Previous studies have demonstrated that the *Wx* gene, which encodes GBSSI and serves as the sole enzyme directly regulating amylose synthesis, plays a pivotal role in governing the AC and GC, thereby acting as the primary determinant of ECQ [9]. The allelic variation of the *Wx* gene is the primary determinant of amylose content disparities among rice varieties. To date, 11 different *Wx* haplotypes and nine mutation sites affecting the function of these genes have been identified (*Wx^a^*, *Wx^b^*, *wx*, *Wx^mp^*, *Wx^op^*, *Wx^mq^*, *Wx^in^*, *Wx^hp^*, and *Wx^lsv^*) [10,11,12,13,14,15,16]. The *Wx^a^* and *Wx^b^* alleles, which are widely distributed in both *indica* and *japonica* rice varieties, correspond to high AC and low AC, respectively, representing two major genetic variations in the rice population. *ALK*/*SSIIa*, near the *Wx* locus on chromosome 6, is the major gene controlling rice GT and is closely associated with rice ECQ [9]. According to the allele variation of the *ALK* gene in Ex8-733 (A/G) and Ex8-864/865 (GC/TT), it is classified into three subtypes based on GC level: *ALK^A-GC^* (A/GC, exhibiting low GC), *ALK^G-GC^* (G/GC, displaying medium-high and high GC), and ALK^G-TT^ (G/TT, demonstrating medium to low GC) [17,18,19]. Current research indicates that *Wx* and *ALK* are the main genes that determine rice quality and are effective target genes for improving rice quality [9]. By using molecular marker-assisted selection, the *Wx^a^* gene of high-yield varieties was replaced with *Wx^b^*, which improved their cooking and taste quality [20]. By combining the molecular markers of *ALK* and backcrossing polymerization of the Minghui 63 variety, the improved rice line achieved a significant reduction in gelatinization temperature, and the appearance quality and cooking taste quality were significantly improved [21].

Rice blast and rice bacterial blight are widely recognized as two prominent diseases that significantly impact rice production, resulting in substantial yield losses and detrimental effects on grain quality [22]. The utilization of resistance genes for the development of durable and broad-spectrum resistant rice varieties represents an effective, cost-efficient, and environmentally sustainable approach to disease prevention. Rice blast, caused by the fungal pathogen *Magnaporthe oryzae* (*Pyricularia oryzae*) [23], is the most devastating rice disease. In the field of genetic research on rice blast resistance, over 100 rice blast resistance alleles have been identified, which are distributed across 69 distinct loci. Among these, over 30 resistance genes have been successfully cloned, including *Pi9*, *Pi2*, *Piz*, *Piz-t*, *Pi40*, *Pi50*, *Pig*, *Pik*, *Pil*, *Pi54*/*Pikh*, *Pik-m*, *Pi-kp*, and *Pigm* [24,25,26]. The resistance gene *Pigm*, derived from Gumei 4, has been widely utilized in rice breeding due to its broad-spectrum and durable resistance to rice blast [27]. The rice *Pigm* locus contains a cluster of genes encoding nucleotide-binding leucine-rich repeat (NLR) receptors that confer durable resistance to the fungus Magnaporthe oryzae without yield penalty. Among these NLR receptors, *PigmR* confers broad-spectrum resistance, whereas *PigmS* competitively attenuates *PigmR* homodimerization to suppress resistance [28]. *Xanthomonas oryzae pv. oryzae* (*Xoo*) causes bacterial blight (BB), a globally devastating disease of rice that is responsible for significant production loss in crops [29]. Additionally, bacterial blight negatively affects rice quality [30]. So far, 15 BB resistance genes have been cloned in rice, including *Xa1*, *Xa2*/*Xa31 (t)*, *Xa3*/*Xa26*, *Xa4*, *xa5*, *Xa7*, *Xa10*, *xa13*, *Xa14*, *Xa21*, *Xa23*, *xa25*, *Xa27*, *xa41 (t)*, and *Xa45 (t)*. The *Xa23* gene executes its function and spectrum of disease resistance by recognizing the cognate TALEs in the pathogen. *Xa23*, derived from Chinese wild rice (*Oryza rufipogon*), has been widely utilized in rice breeding due to its remarkable resistance against various strains of BB throughout the entire growth period [31].

R600 is a heavy-panicle hybrid rice restorer line derived from intersubspecific hybridization of *japonica* and *indica* rice, exhibiting typical traits such as large panicle size, high grain numbers, enhanced lodging resistance, and notable yield superiority. However, the extensive utilization of R600 in hybrid rice combinations is limited by its poor grain quality and susceptibility to rice blast and BB. The aim of this study was to improve grain quality and enhance resistance against rice blast and BB in R600 by pyramiding favorable alleles *Wx^b^*, *ALK^A-GC^*, *Pigm*, and *Xa23*.

## 2. Materials and Methods

### 2.1. Plant Materials

In this study, the heavy-panicle three-line hybrid restorer line R600 was utilized as a recurrent parent for improving grain quality and blast resistance. The line I135, which carries the blast resistance gene *Pigm*, was employed as the donor parent for blast resistance. Similarly, the line I488, harboring the bacterial blight resistance gene *Xa23*, was utilized as the donor parent for bacterial blight. Huanghuazhan (HHZ), an elite rice cultivar with superior quality carrying *Wx^b^* and *ALK^A-GC^* alleles was used as the donor parent for enhancing grain quality. A three-line male sterile line 608A was selected as the female parent to form hybrid combinations. A widely cultivated rice variety in Southwest China, Yixiangyou2115 (YXY2115), was selected as the control for rice quality and production evaluation. The rice varieties LTH and R600 were used as susceptible (S) controls, while I135 and I488 were used as resistant (R) controls.

### 2.2. Population Development and Breeding Selection Procedure

**Figure 1:**
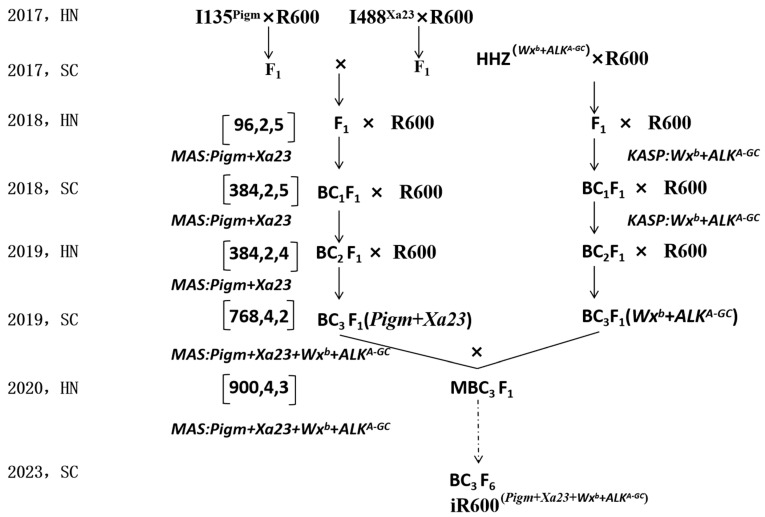
Co-improvement of high quality and disease resistance in R600. R600 was selected as a recurrent parent; I135, I488, and HHZ were chosen as donor parents. The schedule shows the breeding procedure for crossing and backcrossing. To pyramid four target genes in one iR600 line, several successive backcrossing processes were performed, and the backcrossing population was screened to select the desirable individuals. The three digits within the bracket indicate the screening parameters for each backcrossing population, representing the number of individuals evaluated (the first digit), the targeted gene numbers (the second digit), and the selected individuals for backcrossing (the third digit).

The iR600 line was improved via marker-assisted backcrossing as described in Figure 1. To improve the disease resistance of R600, the heavy-panicle restorer line R600 was crossed with two resistance donors I135 (*Pigm*) and I488 (*Xa23*) to generate two distinct F_1_ lines. Subsequently, these two F_1_ lines were crossed to produce double-crossed F_1_ lines, followed by screening for plants containing *Pigm* and *Xa23* genes. The selected double-crossed F_1_ lines were then backcrossed with R600 thrice to obtain BC_3_F_1_ (*Pigm* + *Xa23*) lines. To enhance the quality of R600, a cross was made between R600 and HHZ, followed by two rounds of backcrossing with R600. The BC_3_F_1_ plants harboring *WX^b^* and *ALK^A-GC^* genes were obtained through KASP marker-assisted selection. For each generation of backcrossing, all individual plants were genotyped and only those plants containing the desired target genes underwent subsequent backcrossing and selection. Finally, after six generations of self-pollination, we successfully obtained stable iR600 lines carrying homozygous *Pigm*, *Xa23*, *Wx^b^*, and *ALK^A-GC^* alleles for resistance, grain quality, and agronomic evaluation. The iR600 lines were crossed with 608A, a sterile three-line hybrid, to obtain a hybrid rice combination for comprehensive grain production assessment. All rice plants were cultivated in paddy fields in Chengdu (Sichuan province) and Lingshui (Hainan province).

### 2.3. Marker-Assisted Selection

Two PCR-based InDel markers, *M-Pigm* and *M-Xa23*, were used for selection of resistant genes *Pigm* and *Xa23*. The sequences of *M-Pigm* and *M-Xa23* primers are listed in Table S1. When *M-Pigm* primers were used, the appearance of double bands indicated that the offspring contained the *Pigm* locus of the donor parent I135, while absence of any band indicated a negative result. Meanwhile, when the *Xa23* gene was detected via the *M-Xa23* primer, the 200 bp band indicated that the offspring had transferred the *Xa23* locus from the donor parent I488, while the 300 bp band indicated that it was negative. For genotyping of *Wx* and *ALK* genes, the KASP (Kompetitive Allele Specific PCR)-based molecular markers were designed based on the sequence polymorphism of *Wx* and *ALK* genes between R600 and HHZ parents (Table S2). Specifically, we developed the KASP molecular marker *Wx-2B* to target the C (T/C) nucleotide base of the *Wx* gene, while primer KASP molecular marker *ALK-4A* was designed to detect the G (A/G) nucleotide base of the *ALK* gene. The genotyping assays were tested in a 96-well format and established as 10 μL reactions (2.5 μL of template DNA (50–75 ng), 4.5 μL HiGeno2 × probe Mix, 1.5 μL of primer mix, and 1.5 μL ddH_2_O). PCR was performed on gene amplifiers (TC-96/G/HB) in accordance with the following protocol: hot start at 95 °C for 15 min, 10 cycles (95 ℃ for 20 s and 65 ℃ for 20 s), and then 29 cycles of amplification (94 ℃ for 20 s and 57 ℃ for 60 s), and finally incubation at 37℃ for 1 min.

### 2.4. Genetic Background Analysis

The Affymetrix rice 8K chip, comprising 6933 high-quality SNP markers evenly distributed across the 12 chromosomes of rice, was utilized to compare the genetic background between the improved line iR600 and recurrent parent R600. The total DNA was extracted from leaf samples of 2-week-old rice seedlings. The rice 8K chip assay was performed by China Golden Marker (Beijing, China) Biotechnology Co., Ltd. (Beijing, China) according to the methods previously described [32].

### 2.5. Evaluation of Rice Blast Resistance

The identification of rice blast resistance was conducted in the natural field located in Tiefu Town (Zizhong, China). The local rice blast-induced varieties LTH with high susceptibility to rice blast were employed as control materials for field identification of rice blast. Rice blast-induced varieties were planted around the lines of R600, iR600, and I135. When the LTH rice blast reached full infection (grade 9), the leaf blast phenotype of the test materials was observed, and resistance was determined based on the severity of the leaf blast [33]. For the resistance identification, the materials were randomly divided into three replicates within the designated blast nurseries, and the identification results from different locations were subjected to statistical analysis.

### 2.6. Evaluation of BB Resistance

The hybrid rice restorer line R600, iR600, and I488 (the donor parent for BB) were planted in the field (Chengdu, China) during the summer of 2023 to evaluate their resistance against BB. Inoculation of the *Xanthomonas oryzae pv. oryzae* strain was performed following the method described by Sun [34]. The highly pathogenic strain of rice BB pathogen, *P6* (*PXO99*), was obtained from the National Key Laboratory of Sichuan Agricultural University and stored in a vacuum at −70 °C. The suspension was then used to inoculate the parental line R600 and improve iR600 at the booting stage using the artificial leaf-clipping method. Ten rice plants from each line were selected for testing, and three healthy leaves from each plant were inoculated. The pathogenicity assays on the *Xoo* strain *PXO99* and its derivatives were performed using the leaf-tip-clipping method. Disease symptoms were recorded 2 weeks after inoculation and measured via lesion length [31].

### 2.7. Evaluation of Agronomic Traits

In 2023, parental materials, improved iR600, and test crosses were all planted in the experimental base of experimental fields (Chengdu, China), all according to the standard of 23.33 cm × 16.67 cm. The measurements of the plant height, tiller number, spike length, spike grain per panicle, and other panicle traits were performed at 30 days after heading. For all those traits, ten plants of each line or variety were sampled from each plot, and the main culm of each plant was chosen for trait measurement. At maturity, grain yield per unit area was determined by harvesting forty rice plants, and converting the yield to a per square meter basis. Single plant yield was determined by calculating the total grain yield per plant on ten plants. Panicle length was measured from the neck node to the tip of the main panicle on ten main panicles. The primary branches on the main panicle were counted for ten main panicles. Spikelet density, calculated as the ratio of the total number of grains to the panicle length, was determined for ten panicles. Select 1000 plump grains for weight calculation to represent 1000-grain weight. Setting rate, expressed as a percentage, was determined by comparing the filled grains to the total number of grains for ten panicles. The hybrid combination and control of iR600 focuses on measuring yield, plant height, and tillering. The evaluation method for agronomic traits of hybrid rice combinations is the same as above.

### 2.8. Evaluation of ECQ Quality Traits

Harvested and dried rice grains were kept in a cool place for approximately 30 days to reach moisture equilibrium before processing. Brown rice preparation involved dehusking 100 g of rice using a hulling machine (Experimental rice huller, JLG-2018, Hangzhou, China) to obtain brown rice samples. The brown rice samples were then polished using a whitening machine (Whitening machine, LTJM-2099, Hangzhou, China) to obtain polished rice, which was further sifted using a broken rice separator (Rice separator, FOS-130, Ningbo, China) to obtain whole polished rice. For rice flour preparation, the polished rice was ground into flour using a cyclone mill (Cyclone mill, FS-II, Hangzhou, China), followed by sieving through a 100-mesh sieve to collect the rice flour. Evaluation of rice quality standards in this study was based on the industry standard for eating rice quality (NY/T595-2013) [35].

Chalkiness rate determination was performed on the polished rice using a rice appearance quality judging instrument (Rice appearance quality analyzer, JMWT12, Beijing, China). The length-to-width ratio of grains was read on the same instrument and expressed as a ratio.

Taste score, viscosity, and hardness determinations were performed on 30 g of polished rice cooked with 42 g of water for 30 min. After approximately 2 h, the cooked rice was molded into balls and subjected to taste evaluation using a rice taste analyzer (Rice taste meter, STA1B, New Taipei, Japan) and hardness/viscosity analyzer (Hardness/viscosity analyzer, RHS1A, New Taipei, Japan). AC content determination was conducted using an enzyme-linked immunosorbent assay (ELISA) method on the obtained rice flour, with standard samples obtained from the Chinese Academy of Agricultural Sciences. Data measurements were conducted by Jiangsu Sanshu Biology Co., Ltd. (Nantong, China). GC determination was performed according to the GC method specified in the Chinese national standard (GB/T 22294-2008) for the prepared rice flour [36]. ASV determination was carried out using the ASV method specified in the industry standard (NY 147-88) set by the Ministry of Agriculture and Rural Affairs of China [37].

RVA determination was performed using a Brabender^®^ Micro Visco-Amylo-Graph^®^ (Rapid viscosity analyzer, RVA-Super3, Duisburg, Germany) equipped with a Brabender measuring mixer. The measurement range was set at 300 cmg, with a rotational speed of 250 r/min and unit selection in mPas. The specific procedure involved heating at a rate of 7.5 °C/min up to 95 °C, maintaining the temperature for 5 min, then cooling at 7.5 °C/min down to 50 °C, and finally holding the temperature for 1 min.

### 2.9. Statistical Analysis

Data of grain quality, disease severity, and agronomic traits were presented as the mean ± standard deviation (SD). The *t*-test was performed to examine the statistical significance of differences in all traits by using Microsoft Office Excel software 2010.

### 2.10. Abbreviations

R600: Shuhui 600; BB: bacterial blight; iR600: the improved parental R600 line; ECQ: eating and cooking quality; AC: amylose content; GT: gelatinization temperature; GC: gel consistency; NLR: nucleotide-binding leucine-rich repeat; *Xoo*: *Xanthomonas oryzae pv. oryzae*; HHZ: Huanghuazhan; YXY2115: Yixiangyou2115; KASP: Kompetitive Allele Specific PCR; ASV: alkali spreading value; HD: analysis of head rice; CGP: analysis of chalky grain percentage; CGG: analysis of chalky grain grade; LWR: analysis of length–width ratio; CTS: analysis of comprehensive taste score; AS: analysis of appearance score; TS: analysis of taste score; HPT: analysis of hot paste viscosity; CPT: analysis of cool paste viscosity; BV: analysis of breakdown values; SV: analysis of setback values; CV: analysis of consistency values; YP: yield per plant; TN: tiller number; PH: plant height; SGP: spike grain per panicle; SR: setting rate; SL: spike length; PB: primary branches; SD: spikelet density. MAS: molecular marker-assisted selection; MABC: molecular marker-assisted backcrossing; MAGP: molecular marker-assisted gene pyramiding.

## 3. Results

### 3.1. Development of iR600 Lines by Pyramiding Pigm, Xa23, Wx^b^, and ALK^A-GC^

**Figure 2:**
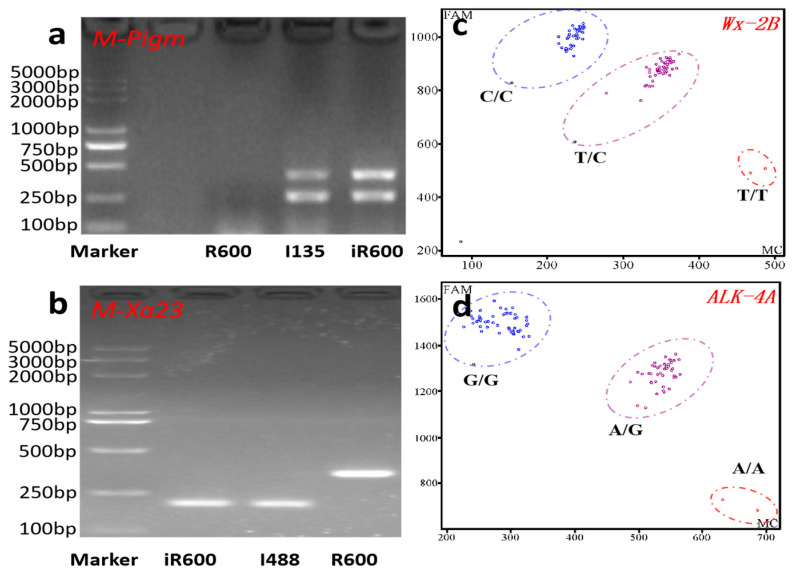
Molecular marker-assisted selection in this study. (**a**) Polymorphism analysis of *Pigm* among recurrent parent R600, donor parent I135, and improved line iR600. The double bands indicated the presence of the *Pigm* locus from the donor parent I135. (**b**) Polymorphism analysis of *Xa23* among R600, I488, and iR600. The band genotype of *Xa23* in I488 and iR600 was significantly smaller than that of R600. (**c**,**d**) KASP genotyping results of *Wx* and *ALK*. The blue circle represents the homozygous allele derived from the donor parent HHZ, while the purple circle signifies heterozygosity, and the red circle denotes alleles originating from R600.

In order to introgress the rice blast resistance gene *Pigm* and the bacterial blight resistance gene *Xa23* into the R600 background, R600 was initially crossed with I135 (donor of *Pigm*) and I488 (donor of *Xa23*), respectively. The F_1_ plants from R600/I135 and R600/I488 were crossed to generate inter-crossing F_1_ lines. Five inter-crossing F_1_ lines harboring both *Pigm* and *Xa23* genes were identified using two PCR-based molecular markers *M-Pigm* and *M-Xa23* (Figure 2a,b) and were subsequently subjected to backcrossing with R600 thrice. In the BC_3_F_1_ generation, four plants with the desired gene combination of *Pigm* and *Xa23*, exhibiting a large panicle size comparable to that of R600, were identified from 384 plants. Simultaneously, the marker-assisted backcrossing between R600 and HHZ (donor parent) was conducted to pyramid desirable quality alleles of *Wx^b^* and *ALK^A-GC^*. Two KASP-based molecular markers, namely *Wx-2B* and *ALK-4A*, were used in this process (Figure 2c,d). In the BC_3_F_1_ generation, individuals with larger panicle size comparable to R600 and a desired heterozygous genotype of *Wx^b^* and *ALK^A-GC^* were obtained.

To further pyramid resistance genes and superior-quality genes, the BC_3_F_1_ (*Pigm* and *Xa23*) and the BC_3_F_1_ (*Wx^b^* and *ALK^A-GC^*) lines were crossed to generate multi-cross F_1_ (MBC_3_F_1_) lines. Through molecular marker detection and field agronomic trait selection, three plants harboring four target genes were selected from 900 MBC_3_F_1_ individuals. These three selected plants exhibited typical architectural characteristics of heavy-panicle rice like R600, such as large panicle size, high grain number, and strong lodging resistance. Furthermore, the subsequent generation MBC_3_F_2_ was obtained through self-pollination. In MBC_3_F_2_ plants, individuals harboring all target genes and exhibiting agronomic traits comparable to or surpassing those of R600 were subsequently subjected to self-pollination to generate MBC_3_F_3_ generations. Through comprehensive evaluation of desired target traits and agronomic characteristics in both background and field screenings, homozygous genotypes of *Pigm* + *Xa23* + *Wx^b^* + *ALK^A-GC^* with superior agronomic traits were continuously selfed until reaching MBC_3_F_6_ (Figure 2). Finally, one stable line from MBC_3_F_6_ with all homozygous target genes and agronomic traits comparable to R600 was successfully obtained and designated as iR600 (Figure 1). To further verify whether the target gene has been introduced into iR600, primers with reported polymorphism of the target gene were selected for validation (Supplementary Table S3) [38,39,40]. The results showed that iR600 did indeed introduce the target gene of the donor material, which differed from the donor (Supplementary Figure S1).

**Figure 3:**
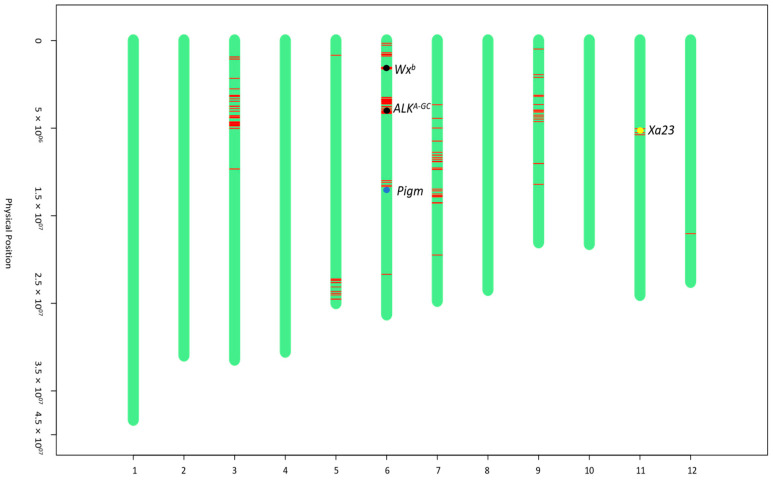
Graphical genotype maps of iR600. The green bars represent the chromosome fragments derived from R600. The red lines represent chromosome fragments derived from donor parents. The black dots indicate the positions of *Wx^b^* and *ALK^A-GC^* genes from the donor parent of HHZ. The blue dot indicates the position of the *Pigm* gene from the donor parent of I135. The yellow dot indicates the position of the *Xa23* gene from the donor parent of I488.

The genetic background analysis of iR600 was conducted using a rice 8K chip containing 6933 evenly distributed SNP markers across all 12 chromosomes (Supplementary Table S4). The results demonstrated a 91.03% recovery rate of the genetic background between iR600 and R600, achieving the anticipated outcomes (Supplementary Table S5). Substitutions of fragments carrying target genes were observed at the positions of *Wx^b^*, *ALK^A-GC^*, and *Pigm* on chromosome 6, as well as *Xa23* on chromosome 11, indicating successful introgression of all four target genes (Figure 3).

### 3.2. Comparison of Grain Quality between iR600 and R600

**Figure 4:**
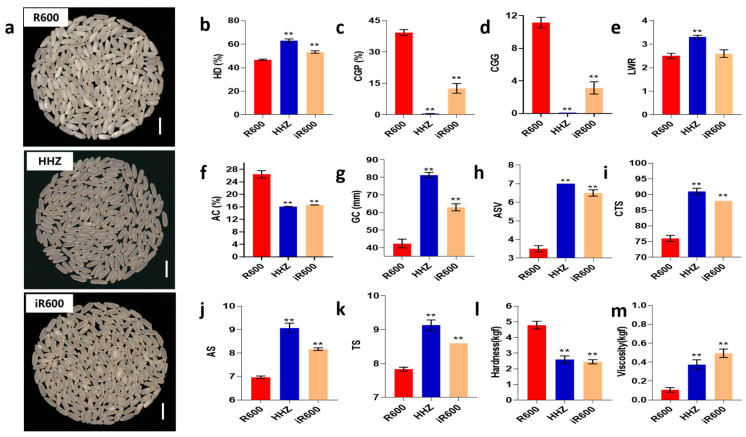
Analysis of grain appearance quality in improved lines. (**a**) The appearance of head rice. Scale bar, 1 cm. (**b**) HD, analysis of head rice (*n* = 3). (**c**) CGP, analysis of chalky grain percentage (*n* = 3). (**d**) CGG, analysis of chalky grain grade (*n* = 3). (**e**) LWR, analysis of length–width ratio (*n* = 10). (**f**) AC, analysis of amylose content (*n* = 3). (**g**) GC, analysis of gel consistency (*n* = 3). (**h**) ASV, analysis of alkali spreading value (*n* = 3). (**i**) CTS, analysis of comprehensive taste score (*n* = 3). (**j**) AS, analysis of appearance score (*n* = 3). (**k**) TS, analysis of taste score (*n* = 3). (**l**) Analysis of hardness (*n* = 3). (**m**) Analysis of viscosity (*n* = 3). The significant comparison object is R600. A two−tailed Student’s t-test was used to generate *p*-values. ** indicate a significant difference at *p* < 0.01; Values are means ± SD. Error bars represent SDs.

To determine whether iR600 achieved the grain quality characteristics, we first compared the grain appearance quality to that of its parent R600. We observed that the iR600 lines exhibited a superior grain appearance compared to R600 and were similar to the donor parent HHZ (Figure 4a). The head rice rate of the improved line iR600 was significantly higher than that of R600, but it was still lower than that of HHZ (Figure 4b). The chalky grain percentage of milled rice in iR600 decreased significantly compared to the original parent R600, accompanied by a reduction in chalky grain grade and a significant increase in head rice (Figure 4c,d). The length–width ratio of the grains in iR600 was slightly greater than that in R600, although the difference is not statistically significant (Figure 4e). We assessed ECQ by quantifying three physicochemical properties AC, GC, and alkali spreading value (ASV, the index for the gelatinization temperature). The results showed that compared with R600, the three physicochemical properties of iR600 were significantly improved, approaching the high-quality level of superior quality parent HHZ (Figure 4f–h). In detail, the iR600 lines exhibited a significant decrease in amylose content, a notable improvement in ASV, and a substantial increase in gel consistency levels. The iR600 lines also demonstrated a significant enhancement in the ECQ of cooked rice, exhibiting a remarkable improvement in the comprehensive taste score, appearance score, and taste score (Figure 4i,m). In summary, the improved line iR600, which pyramided the *Wx^b^* and *ALK^A-GC^* alleles from HHZ, exhibited significantly enhanced grain quality compared to R600.

### 3.3. The RVA Profile of iR600 Starch

**Figure 5:**
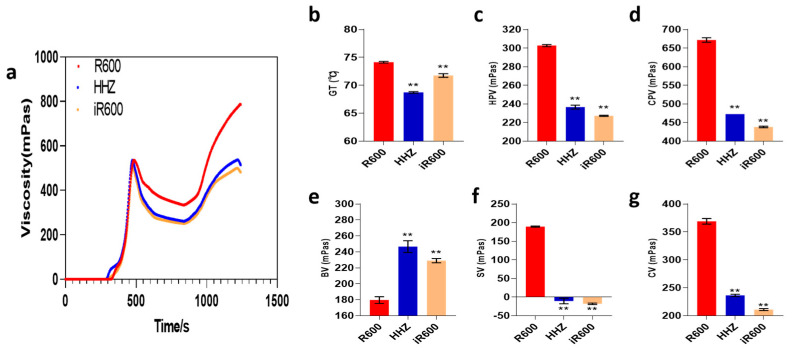
The RVA characteristic curves of improved lines. (**a**) The RVA characteristic curves. (**b**) GT, analysis of gelatinization temperature (*n* = 3). (**c**) HPT, analysis of hot paste viscosity (*n* = 3). (**d**) CPT, analysis of cool paste viscosity (*n* = 3). (**e**) BV, analysis of breakdown values (*n* = 3). (**f**) SV, analysis of setback values (*n* = 3). (**g**) CV, analysis of consistency values (*n* = 3). The significant comparison object is R600. A two−tailed Student’s t-test was used to generate *p*-values. ** indicate a significant difference at *p* < 0.01. Values are means ± SD. Error bars represent SDs.

Starch is the main component of rice and plays a crucial role in determining rice quality. To elucidate the specific impact of improvement on starch viscosity and thermodynamic properties, we conducted RVA analysis in the parental lines R600 and HHZ, as well as the improved line iR600. The RVA curve of iR600 exhibited a close overlap with that of donor parent HHZ, while significantly differing from the RVA curve of its recurrent parent R600 (Figure 5a). We further statistically analyzed the six RVA-associated physicochemical properties in iR600, comparing them to those of R600 and HHZ. iR600 exhibited a lower gelatinization temperature compared to R600, indicating that the introduction of the *ALK^A-GC^* allele from HHZ effectively reduced the gelatinization temperature (GT) of iR600 (Figure 5b). Notably, the hot paste viscosity (HPV), cool paste viscosity (CPV), breakdown values (BDV), setback values (SBV), and consistency values (CSV) of iR600 exhibited remarkable alterations compared to the original parent R600 (Figure 5c–g). All the six RVA-associated physicochemical properties in iR600 were close to the donor parent HHZ, indicating that the introduction of the major grain quality controlling locus *Wx^b^* and *ALK^A-GC^* effectively improved its starch traits.

### 3.4. Evaluation of iR600 Resistance to Rice Blast and BB

**Figure 6:**
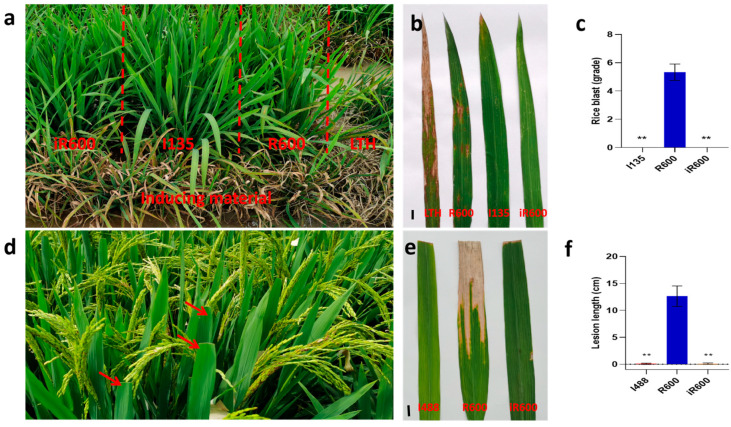
Evaluation and analysis of disease resistance of improved lines. (**a**) Rice blast is naturally induced. (**b**) Incidence of leaf blast. Scale bar, 1 cm. (**c**) Disease analysis of rice blast (*n* = 3). (**d**) Bacterial blight inoculation. (**e**) Incidence of bacterial blight. Scale bar, 1 cm. (**f**) Analysis of leaf blight spot length (*n* = 10). The significant comparison object is R600. The red arrow represents the inoculated leaves. A two−tailed Student’s *t*-test was used to generate *p*-values. ** indicate a significant difference at *p* < 0.01. Values are means ± SD. Error bars represent SDs.

The field resistance of rice blast assessment was conducted in Zizhong County, Sichuan Province in 2023. The donor parent I135 and the improved line iR600, carrying the *Pigm* gene, both exhibited a high level of resistance to rice blast disease at the seedling stage with zero disease score. In contrast, the recurrent parent R600 demonstrated high susceptibility with a disease score of 5.33 (Figure 6a–c). In order to assess the resistance of BB, we measured the average lesion length caused by *Xoo* strain *P6* after 14 days of inoculation. The resistance assays revealed that R600 exhibited susceptibility to BB, with an average lesion length of 12.63 cm. Conversely, both the donor parent I488 and iR600, which carried the *Xa23* gene, displayed minimal symptoms on leaves with a mean lesion length of 0.09 cm and 0.11 cm, respectively, indicating comparable levels of BB resistance between iR600 and I488 (Figure 6d–f). These results clearly demonstrated that the introduction of *Pigm* and *Xa23* genes significantly enhanced the rice blast and BB resistance in iR600.

### 3.5. Agronomic Trait Performance of iR600

**Figure 7:**
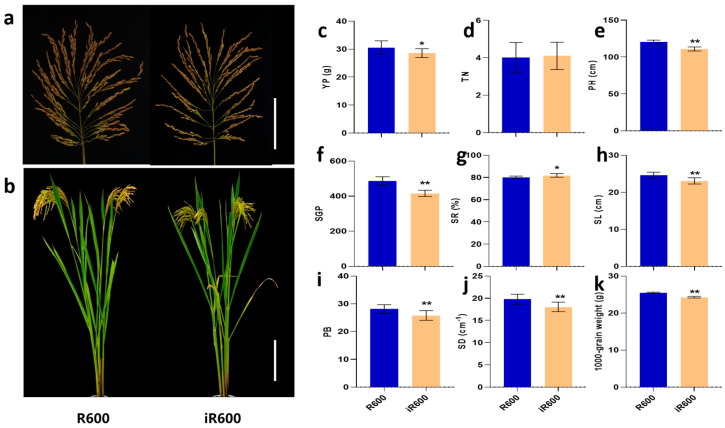
Evaluation of main agronomic characters in improved lines. (**a**) Main panicles. Scale bar, 10 cm. (**b**) Plant morphology. Scale bar, 20 cm. (**c**) YP, yield per plant (*n* = 10). (**d**) TN, tiller number (*n* = 10). (**e**) PH, plant height (*n* = 10). (**f**) SGP, spike grain per panicle (*n* = 10). (**g**) SR, setting rate (*n* = 10). (**h**) SL, spike length (*n* = 10). (**i**) PB, primary branches (*n* = 10). (**j**) SD, spikelet density (*n* = 10). (**k**) 1000-grain weight (*n* = 10). The significant comparison object is R600. A two−tailed Student’s *t*-test was used to generate *p*-values. * and ** indicate a significant difference at *p* < 0.05 and 0.01. Values are means ± SD. Error bars represent SDs.

The main agronomic traits of iR600 were evaluated compared with the original parent R600. In general, the improved line iR600 consistently exhibited similar heavy-panicle traits to the recurrent parent R600, showing a large panicle size (23.09 cm) and high spike number per panicle (416.40), and basically retained the overall architecture characteristics of R600 (Figure 7a,b,f,h). The panicle length, spike number per panicle, and spike density of iR600 were observed to be 6.25%, 14.46%, and 8.70% lower than those of R600; however, the seed-setting rate of iR600 exhibited a higher value than that of R600 (Figure 7f,h,j). There was no significant difference in tiller number between iR600 and R600, but the plant height of iR600 was significantly lower than that of R600, which was beneficial to the enhancement of lodging resistance (Figure 7d,e). We then conducted a field experiment to investigate the yield per plant of iR600 and R600, which revealed that iR600 exhibited only a 6.58% reduction in comparison to R600 (Figure 7c). These results indicated that the introgression of *Pigm*, *Xa23*, *Wx^b^*, and *ALK^A-GC^* alleles from the donor parents had minimal effects on the yield-related traits in iR600; however, it significantly improved the grain quality and disease resistance while retaining the characteristics of heavy-panicle in iR600 (Figure 7).

### 3.6. Application of iR600 in Hybrid Rice Breeding

**Figure 8:**
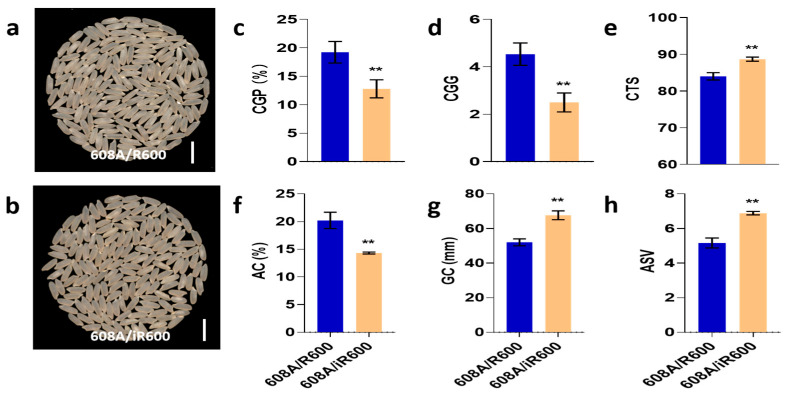
Analysis of rice quality in hybrid combinations. (**a**) Whole-milled rice appearance of 608A/R600. Scale bar, 1 cm. (**b**) Whole-milled rice appearance of 608A/iR600. Scale bar, 1 cm. (**c**) CGP, chalky grain percentage of hybrid combinations (*n* = 3). (**d**) CGG, chalky grain grade of hybrid combinations (*n* = 3). (**e**) CTS, comprehensive taste score of hybrid combinations (*n* = 3). (**f**) AC, amylose content of hybrid combinations (*n* = 3). (**g**) GC, gel consistency of hybrid combinations (*n* = 3). (**h**) ASV, alkali spreading value of hybrid combinations (*n* = 3). The significant comparison objects are the cross combinations of R600 corresponding to male sterile line. A two-tailed Student’s *t*-test was used to generate *p*-values. ** indicate a significant difference at *p* < 0.01. Values are means ± SD. Error bars represent SDs.

To explore the application of iR600 in hybrid rice breeding, we crossed iR600 and R600 with a high-quality three-line sterile line 608A, respectively, and compared the comprehensive performance of hybrid combinations. The resulting hybrid combinations of iR600/608A and R600/608A were then compared in field plot experiments to assess their comprehensive performance. The comparison of grain quality revealed a noticeable improvement in the appearance quality of iR600/608A compared to R600/608A with a significant decrease observed in both the rate of chalky grain percentage and the chalky grain grade (Figure 8a–d). The iR600/608A hybrids exhibited significant improvements in terms of amylose content, gel consistency, ASV, and taste index compared to the R600/608A hybrids (Figure 8e–h), achieving a high-quality level 2 according to the edible rice variety quality standards (NY/T595-2013).

**Figure 9:**
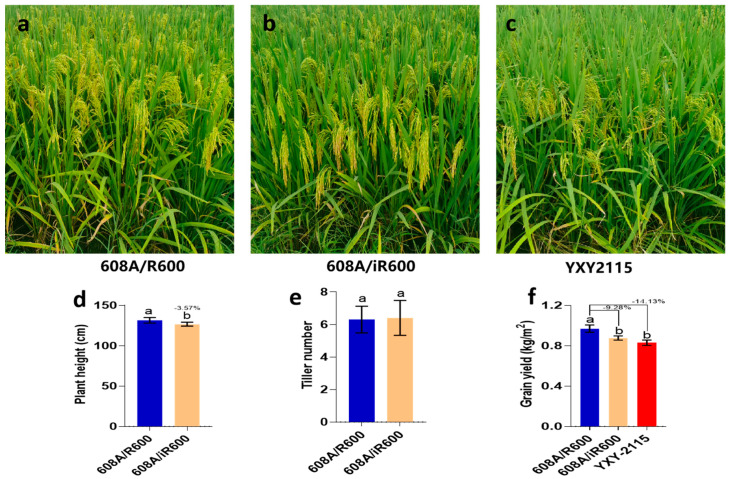
Major agronomic phenotypes of hybrid combinations. (**a**–**c**) Field growth of hybrid combinations. (**d**) Yield of hybrid combination (*n* = 3). (**e**) Plant height of hybrid combination (*n* = 10). (**f**) Tiller number of hybrid combinations (*n* = 10). Values are means ± SD. Error bars represent SDs. Different lowercase letters indicate significant differences (*p* ≤ 0.05; one−way ANOVA).

To evaluate the yield potential of iR600/608A, a comparative analysis was conducted on the yield and key agronomic traits of iR600/608A, R600/608A, and YXY2115, which is a widely cultivated rice variety in southwest China. The results showed that iR600/608A had excellent plant morphology and inherited the characteristics of a typical large panicle (Figure 9a,b). In comparison to R600/608A, iR600/608A exhibited a significant reduction in plant height by 3.57%, while no discernible difference was observed in tiller number (Figure 9d,e). The yield of iR600/608A was reduced by 9.28% compared to R600/608A, which is comparable to the widely cultivated rice variety YXY2115 (Figure 9f). These results indicated that the hybrid rice derived from the improved iR600 exhibited evident quality advantages compared to R600.

## 4. Discussion

Over the past few decades, the heavy-panicle type of hybrid rice has demonstrated remarkable yield and extensive adaptability in the southwestern region of China. However, its widespread promotion is impeded by limitations concerning rice quality and disease resistance. Due to the lengthy breeding cycle and limited efficacy of conventional breeding methods, it poses a challenge to simultaneously enhance rice quality and disease resistance while preserving the high yield potential of heavy-panicle rice parents. In recent years, the identification of a substantial number of key functional genes and the update of genomic sequences in rice have significantly advanced the practical application of molecular breeding technology, including molecular marker-assisted selection (MAS), molecular marker-assisted backcrossing (MABC), and molecular marker-assisted gene pyramiding (MAGP) [41]. Rational design is a powerful strategy for pyramiding multiple complex traits in modern rice breeding [38]. In this study, four beneficial alleles (*Wx^b^*, *ALK^A-GC^*, *Pigm*, and *Xa23*) were successfully pyramided in the heavy-panicle hybrid rice restorer line R600 through MABC using four effective molecular markers. The resulting iR600 line, with a genetic background recovery rate of 91.03%, exhibited superior grain quality and enhanced disease resistance compared to the original parent R600. These findings have significant practical implications for enhancing multiple traits in heavy-panicle hybrid rice breeding.

Utilizing broad-spectrum durable resistance for rice improvement is widely acknowledged as the most cost-effective and efficient approach to mitigate yield losses caused by diverse diseases [42]. Therefore, the integration of major genes exhibiting broad-spectrum and durable resistance against diverse diseases represents a highly effective breeding strategy for enhancing disease resistance [43]. Numerous studies have demonstrated the high breeding value of *Pigm* in developing rice varieties with durable and broad-spectrum resistance to both seedling and panicle blast [28,44]. Our results showed that the iR600 line carrying the *Pigm* allele exhibited significantly higher resistance to blast disease than the recurrent parent R600 under natural infection conditions in the blast nursery. The *Xa23* gene, known for its dominant effect and robust resistance to BB, has been widely utilized in breeding programs due to its broad spectrum of resistance [45]. Consistent with expectations, the improved line iR600 containing *Xa23* exhibited a high resistance to the virulent *Xoo* strain *P6*. These results indicate that the introduction of *Pigm* and *Xa23* simultaneously enhances the resistance of R600 to rice blast and BB, thereby validating the effectiveness of this breeding strategy in improving disease resistance in heavy-panicle hybrid rice breeding.

ECQ is the most widely concerned rice quality trait by consumers, breeders, and researchers [46]. It is widely believed that rice cultivars with low-to-intermediate amylose content (AC) exhibit superior palatability, stickiness, and hardness in comparison to those with high-AC [9]. In addition, the gelatinization temperature (GT) is also regarded as a crucial indicator for assessing the quality of rice ECQ [47]. The major genes controlling AC and GT in rice are *Wx and ALK,* respectively. In modern rice breeding programs, the utilization of elite alleles such as *Wx^b^* for medium-low AC and *ALK^A-GC^* for low GT has been a predominant strategy to improve rice ECQ [48,49]. We found that the heavy-panicle restorer line R600 with genotypes of *Wx^a^* and *ALK^G-GC^* showed high AC and low GT, resulting in poor ECQ. To improve the ECQ of R600, the elite alleles of *Wx^b^* and *ALK^A-GC^* from donor line HHZ were introduced into R600 through MAS, utilizing two functional KASP markers, *Wx-2B* and *ALK-4A*, designed based on the polymorphism of *Wx* and *ALK* haplotypes between R600 and HHZ. The obtained line iR600, carrying *Wx^b^* and *ALK^A-GC^*, not only exhibited decreased AC and GT but also conferred numerous favorable effects on ECQ, including improved grain appearance, increased GC, and higher taste score. Moreover, the hybrid combination of iR600/608A also demonstrated superior quality and high yield potential, suggesting that iR600 holds a great application value in breeding high-quality heavy-panicle hybrid rice.

The objective of modern breeding is to develop varieties that with high yield, superior quality, desired architecture, and enhanced resistance [50]. However, the simultaneous enhancement of multiple traits poses a challenge due to the inherent “trade-off” between different traits [43,51]. The activation of immune responses through resistance genes to combat pathogens can sometimes result in a decrease in other agronomic traits, leading to a yield penalty known as the growth–defense trade-off [52]. In this study, we developed iR600 by incorporating four elite alleles (*Pigm*, *Xa23*, *Wx^b^*, and *ALK^A-GC^*) from three different donor parents into the heavy-panicle hybrid restorer line R600. Although iR600 demonstrated excellent quality and high disease resistance, its yield was somewhat compromised compared to the recurrent parent R600, with a decrease observed in both panicle size and grain numbers. We also found that although the iR600 hybrid combination of iR600/608A exhibited a superior yield level, surpassing the current large-scale promotion of the YXY2115 variety in southwest China, it still resulted in a 9.28% reduction in yield compared to the original R600/608A hybrid combination. It is hypothesized that the pyramid of two dominant *R* genes, *Pigm* and *Xa23*, in iR600 confers resistance against rice blast and bacterial blight on both the restorer line itself and its hybrid rice progeny, while potentially exerting a penalty on panicle development, leading to a reduction in yield.

In this study, although we used four molecular markers tightly linked to each target gene for foreground selection throughout the breeding process, we did not select the genetic background in each generation but instead screened the target lines based on field agronomic traits. The rice 8K chip assay revealed that the background recovery rate between iR600 and R600 is 91.03%, suggesting that the presence of non-target genomic fragments and unexpected inheritance linked by target genes from the three donor parents may lead to a penalty in yield-related traits in iR600. Despite a slight reduction in yield, iR600 still exhibits high yield potential and demonstrates superior characteristics in terms of rice quality and resistance, presenting a broader application prospect in hybrid rice breeding. The iR600 line still exhibited typical heavy-panicle traits, with nearly 400 grains per panicle, demonstrating significant yield potential in hybrid combinations. Moreover, it possesses high disease resistance and excellent quality, making it highly valuable for future breeding programs focused on heavy-panicle hybrid rice.

## 5. Conclusions

In summary, we successfully developed a new elite heavy-panicle hybrid restorer line by pyramiding major genes (*Wx^b^* + *ALK^A-GC^* + *Pigm* + *Xa23*) that significantly contribute to grain quality and disease resistance. The newly bred line iR600 exhibited superior rice quality and demonstrated high resistance against rice blast and BB, while maintaining typical heavy-panicle traits and displaying strong heterosis. The present study demonstrates that the simultaneous enhancement of rice quality and resistance can be achieved through the introduction of major genes via molecular design, providing valuable insights for enhancing parental traits in heavy-panicle hybrid rice.

## Data Availability

All data reported in this manuscript were obtained during this study and the data are included in the manuscript fles as well as in the Supplementary Materials.

## Supplementary Materials

The following supporting information can be downloaded at: https://www.mdpi.com/article/10.3390/cimb46100639/s1, Figure S1: Polymorphism of designed markers among R600, iR600, I135 and I488; Table S1: Primers of PCR-based InDel markers; Table S2: Primers of KASP-based SNP markers; Table S3: Primers used to detect polymorphisms of targeted genes in this study; Table S4: Information on Rice 8K Chip in R600; Table S5: Information on Rice 8K Chip in iR600.
