# Supplementary figures and images for: Improvement of Quality and Disease Resistance for a Heavy-Panicle Hybrid Restorer Line, R600, in Rice (Oryza sativa L.) by Gene Pyramiding Breeding
Source: Curr Issues Mol Biol. 2024 Sep 25;46(10):10762–78. doi: 10.3390/cimb46100639 (PMC11505696; doi:10.3390/cimb46100639)

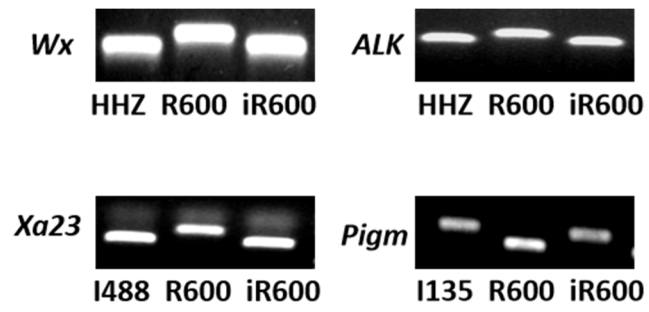

**Supplementary Figure S1.** Polymorphism of designed markers among R600, iR600, I135 and I488.

Supplement: Supplementary file 1 [file cimb-46-00639-s001.zip › Supplementary Figure S1. Polymorphism of designed markers among R600, iR600, I135 and I488.pdf]
